# LLM-as-a-Judge: automated evaluation of search query parsing using large language models

**DOI:** 10.3389/fdata.2025.1611389

**Published:** 2025-07-21

**Authors:** Mehmet Selman Baysan, Serkan Uysal, İrem İşlek, Çağla Çığ Karaman, Tunga Güngör

**Affiliations:** ^1^sahibinden.com, Istanbul, Türkiye; ^2^sahibinden.com, Ankara, Türkiye; ^3^Department of Computer Engineering, Faculty of Engineering, Boğaziçi University, Istanbul, Türkiye

**Keywords:** LLM-as-a-Judge, structured output evaluation, search query parsing, large language models, evaluation framework, generative search, automatic evaluation, query understanding

## Abstract

**Introduction:**

The adoption of Large Language Models (LLMs) in search systems necessitates new evaluation methodologies beyond traditional rule-based or manual approaches.

**Methods:**

We propose a general framework for evaluating structured outputs using LLMs, focusing on search query parsing within an online classified platform. Our approach leverages LLMs' contextual reasoning capabilities through three evaluation methodologies: Pointwise, Pairwise, and Pass/Fail assessments. Additionally, we introduce a Contextual Evaluation Prompt Routing strategy to improve reliability and reduce hallucinations.

**Results:**

Experiments conducted on both small- and large-scale datasets demonstrate that LLM-based evaluation achieves approximately 90% agreement with human judgments.

**Discussion:**

These results validate LLM-driven evaluation as a scalable, interpretable, and effective alternative to traditional evaluation methods, providing robust query parsing for real-world search systems.

## 1 Introduction

The adoption of large language models (LLMs) in search systems is fundamentally reshaping how these systems function, driving the emergence of generative search beyond traditional retrieval methods. This shift introduces new challenges in the evaluation of search system performance, especially in real-world applications such as online classified ads platforms, where accurately interpreting user search queries is essential to improve the retrieval and ranking quality (Luo et al., 2023).

**Figure 1:**
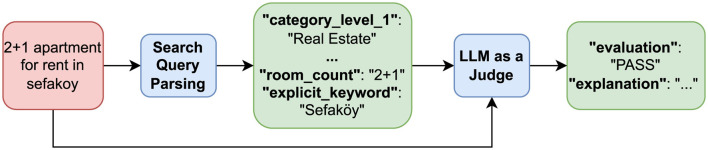
Overview of the LLM-as-a-Judge evaluation process for a sample search query.

Figure 1 shows the parsing of an example search query in an online ads platform and a sketch of the evaluation process proposed in this work. The search system extracts structured information from the user query given in natural language, including query category, search filters, location, explicit and implicit keywords, synonyms, and other relevant attributes. The extracted elements are represented in a structured form which is used as the basis for evaluation. Unlike traditional syntactic parsing, search systems parse based not only on the textual features of the query but also on its semantics and contexts. This implies that the system infers implicit intentions, resolves ambiguities, and maps the query to a structured representation that aligns with its underlying meaning rather than just its surface form.

Traditional evaluation methods for search query parsing such as exact match, precision, recall, and rule-based heuristics like the number of search results returned often struggle to fully reflect the query understanding capability of the systems used in complex applications (Lee et al., 2021). Additionally, these approaches may not effectively capture the semantic nuances of the parsed user queries, leading to limited generalizability. Task-specific evaluation methods, such as checking whether a query retrieves the correct results or measuring how users interact with search results, can provide better assessments. However, these methods are highly dependent on domain specific rules and are difficult to apply across different search systems, making them less flexible (Jiang and Cai, 2024). Moreover, manual evaluation methods, which involve human annotators assessing query parsing results, are both time-consuming and expensive, making them impractical for large-scale, real-time systems. Due to the ambiguity inherent in natural language, there is often no single correct output for a given query, as multiple valid structured representations may exist depending on subtle differences in context and user intent. Traditional evaluation methods rely on rigid comparisons against predefined reference outputs and thus cannot handle cases where multiple outputs are equally valid or where errors are subtle yet impactful. Consequently, a more context-aware and flexible evaluation approach is necessary to assess the effectiveness of the search system in understanding and structuring user queries.

The challenge is particularly evident in high-traffic environments such as e-commerce platforms, where the ability to accurately parse user queries into structured representations is crucial for delivering relevant search results. In these scenarios, traditional evaluation methods face difficulties in scaling to the vast number of user queries, ensuring real-time adaptability, and mitigating inconsistencies in human-labeled reference datasets. Beyond these commonly-used methods, other automated evaluation techniques, such as BLEU (Papineni et al., 2002) for n-gram overlap or Smatch (Cai and Knight, 2013) for Abstract Meaning Representation (AMR), have shown promise but cannot fully address the complexity of evaluating structured outputs like search query parsing results.

The LLM-as-a-Judge framework initially emerged as a promising approach for evaluating various natural language processing (NLP) tasks, providing an automated and scalable alternative to human assessments. Over time, its application has expanded to include the evaluation of structured outputs of various systems. While still an evolving method, it offers the potential for more scalable and consistent assessments compared to traditional techniques. Recent research (Schneider et al., 2024) has demonstrated that LLMs can effectively evaluate semantic parsing tasks by leveraging their ability to understand natural language nuances and assess the correctness of structured outputs beyond surface-level lexical matching.

In this work, we employ automated LLM-based evaluation methods for assessing search query parsing systems, which leverage the reasoning and contextual understanding capabilities of LLMs. Unlike rule-based or heuristic evaluation methods, which may fail to generalize across different query formulations, LLMs offer a more nuanced and adaptable evaluation framework. These methods enable the system to be assessed not just on surface-level correctness but also on semantic fidelity, intent alignment, and contextual appropriateness. By integrating LLM-as-a-Judge methods, we ensure a more robust and scalable evaluation of search query parsing in large-scale, real-world search systems, where accuracy and efficiency are critical. However, integrating LLMs into evaluation pipelines introduces new complexities, necessitating careful design to ensure reliability, efficiency, and alignment with human judgment. In this work, we explore strategies to mitigate these challenges, refining LLM-based evaluations to enhance consistency and reduce hallucinations, ultimately making them a viable alternative to both costly and labor-intensive human evaluations and evaluations made by traditional syntax-based/word-based metrics.

We explore three distinct LLM-as-a-Judge methodologies: *Pointwise Evaluation, Pairwise Evaluation*, and *Pass/Fail Evaluation*. Our approach builds upon existing research in semantic parsing evaluation, incorporating elements from both traditional metrics like Smatch and newer LLM-based assessment techniques. We conduct extensive experiments using various LLMs, prompting strategies, and evaluation techniques, and demonstrate that our LLM-as-a-Judge framework achieves over 90% alignment with human judgments across evaluation types. Furthermore, we introduce a *Contextual Evaluation Prompt Routing* strategy within LLM evaluation to enhance the efficiency of the evaluation and mitigate LLM hallucinations. Our findings validate the effectiveness of LLM-driven automated evaluation for search query parsing in large-scale, real-world search systems, offering a scalable and adaptable evaluation pipeline that minimizes manual effort. Beyond structured output evaluation, we also examine the reliability of our LLM evaluator framework using statistical agreement metrics to ensure the robustness of LLM-based assessments.

The contributions in this work are as follows:

- We propose an evaluation framework that leverages large language models for context-aware, interpretable, and scalable evaluation of structured outputs.
- We apply the proposed evaluation framework to search query parsing by adapting the Pointwise, Pairwise, and Pass/Fail evaluation strategies to address various assessment requirements.
- We introduce the Contextual Evaluation Prompt Routing strategy as a domain-specific solution for dynamically adjusting evaluation prompts based on query categories, enabling more accurate and context-aware assessment of structured search query parsing outputs.
- We show that the proposed framework, particularly the Contextual Evaluation Prompt Routing strategy, substantially improves the evaluation accuracy and reliability compared to baseline methods.

## 2 Related work

The evaluation of search query parsing and semantic parsing systems has been a longstanding challenge in NLP and information retrieval (IR). Various methods ranging from rule-based systems to neural models have been proposed to improve parsing accuracy and assessment. Recently, LLM-as-a-Judge has emerged as a promising approach for evaluating structured outputs, offering scalability and adaptability across different domains. In this section, we review traditional evaluation methods for search query parsing, discuss general LLM-as-a-Judge techniques, and explore domain-specific applications of LLM-as-a-Judge, particularly in search and semantic parsing systems.

### 2.1 Traditional evaluation methods

Traditional evaluation methods for search query parsing and semantic parsing rely on exact match accuracy, precision-recall metrics, and rule-based heuristics. While these approaches measure the correctness by comparing system outputs to reference query outputs, they often fail to capture semantic equivalence, penalizing valid variations in structured outputs (Lee et al., 2021). Early methods assessed correctness through query execution accuracy, where system-generated queries are executed against a database and the returned results determine the accuracy (Jiang and Cai, 2024). While this approach is applicable in search query parsing, evaluating the correctness of the results still requires manual judgments. Luo et al. (2023) introduced precision and coverage metrics to evaluate attribute extraction, but these methods require manual judgments, making them less scalable.

With the rise of neural semantic parsers, evaluation techniques increasingly incorporated denotation-based methods while retaining other approaches. Denotation-based evaluation, which compares execution results rather than output structures, was already used in non-neural settings and gained further prominence with neural models. Additionally, though initially designed for machine translation, statistical metrics like BLEU (Papineni et al., 2002) continued to be used for assessing semantic parsing outputs. However, these approaches face some challenges:

- Spurious matches—Incorrect queries may produce correct results by chance.
- Over-penalization—Semantically correct but syntactically different outputs are unfairly penalized.
- Lack of semantic awareness—BLEU and similar metrics fail to capture deep semantic understanding.

Graph-based evaluation metrics like Smatch (Cai and Knight, 2013) attempt to address these issues by measuring semantic structure similarity rather than strict string matching. While effective, these methods are computationally expensive and not widely used in search query parsing.

Overall, traditional evaluation methods struggle with generalization, adaptability, and deeper semantic reasoning. This highlights the need for more semantically oriented evaluators, which can assess semantic correctness beyond surface-level comparisons. Our work builds on these findings by employing LLM-as-a-Judge to evaluate structured search query parsing, ensuring alignment with user intent and domain-specific accuracy.

### 2.2 LLM-as-a-Judge for automated evaluation

LLM-as-a-Judge has gained traction as a scalable alternative to human evaluations across various NLP tasks, including text summarization, dialogue evaluation, and semantic parsing. The concept leverages the reasoning capabilities of large language models to assess system outputs, reducing reliance on costly human annotations while maintaining evaluation consistency and scalability.

A comprehensive survey by Gu et al. (2025) explores how LLM-as-a-Judge can enhance evaluation reliability by addressing challenges such as bias mitigation, prompt engineering, and standardization of evaluation methodologies. The study highlights that LLMs offer more nuanced assessments compared to rule-based or heuristic metrics, particularly in tasks requiring semantic alignment rather than syntactic matching. Similarly, Li H. et al. (2024) provide a structured framework for constructing LLM-based evaluation pipelines, addressing how LLMs can be utilized effectively, where they perform best and how they should be assessed.

Another critical aspect is the evaluation process itself. Chiang and Lee (2023) analyze different strategies, revealing that forcing LLMs to output only a single numeric rating is suboptimal, while prompting LLMs to explain their ratings significantly improves alignment with human judgments. These findings emphasize the importance of prompt engineering and structured evaluation prompts to enhance the reliability of LLM-generated assessments.

Despite their advantages, LLM-based evaluators are prone to stochastic variability, position bias, verbosity bias, and self-enhancement bias. Zheng et al. (2023) highlight these limitations in their study on MT-Bench and Chatbot Arena, demonstrating that while LLM judges such as GPT-4 align with human preferences over 80% of the time, they still require bias mitigation techniques to ensure fair assessments. To address reliability concerns, researchers have explored adaptive evaluation techniques. Shankar et al. (2024) introduce EvalGen, a system that refines evaluation prompts through human-in-the-loop feedback. They identify criteria drift, where evaluation criteria evolve as human reviewers assess more outputs. This aligns with our prompt engineering with iterative refinement approach, which systematically optimizes evaluation prompts to enhance consistency and reduce hallucinations. In our experiments, since we use evaluators from the same model family we do not consider self-enhancement bias a significant threat. However, position bias and stochastic variability remain critical challenges in our context. This study explicitly addresses these two sources of evaluation instability through experimental controls such as randomized response ordering and repeated runs with majority voting or averaging to enhance robustness.

A major challenge in LLM-based evaluation is result consistency across multiple replications. Schroeder and Wood-Doughty (2024) introduce McDonald's omega as a measure of evaluation reliability, assessing how sensitive LLM evaluators are to small variations in input conditions. Their study emphasizes that single-shot evaluations may introduce inconsistencies, reinforcing the need for multiple evaluation iterations and statistical reliability measures, which is an approach we integrate into our study.

While these studies focus on LLM-based evaluation across general NLP tasks, their insights inform our approach to evaluating structured outputs in search query parsing. Our study extends this paradigm by adapting LLM-as-a-Judge to structured JSON-like evaluations, ensuring that search query parsing accuracy is assessed through semantic correctness, intent alignment, and contextual appropriateness rather than surface-level comparisons.

### 2.3 Domain-specific LLM-as-a-Judge and its application in search query parsing

While LLM-as-a-Judge has been widely explored in general text generation and evaluation tasks, its application in domain-specific structured evaluation, such as search query parsing and semantic parsing, presents additional challenges. Unlike free-text evaluation, query parsing evaluation requires assessing structured outputs, including logical forms, database queries, or graph representations. Recent research has focused on adapting LLM-based evaluation frameworks to domain-specific tasks, ensuring that evaluations align with business needs, structured information retrieval, and reasoning-intensive applications.

A key challenge in domain-specific LLM-based evaluation is the need for custom evaluation criteria. Zhang et al. (2024) introduce TALEC, a model-based evaluation method that enables users to flexibly define their own evaluation criteria based on domain requirements. Their approach leverages zero-shot and few-shot in-context learning (ICL) to teach LLMs in-house evaluation rules, improving adaptability across different business scenarios. By combining prompt engineering with iterative refinement, TALEC achieves over 80% correlation with human judgments, demonstrating that LLM-as-a-Judge can accurately reflect domain-specific quality standards. Our work builds upon these findings by applying the Contextual Evaluation Prompt Routing strategy to search query parsing evaluation, where domain-specific prompts are dynamically selected based on query categories, ensuring that evaluation criteria remain contextually relevant.

Another critical aspect of LLM-based evaluation in domain-specific applications is the construction of specialized evaluation datasets. Raju et al. (2024) propose a data pipeline for curating domain-specific evaluation sets, addressing the limitations of general-purpose benchmarks like AlpacaEval 2.0 LC (Dubois et al., 2024) and Arena-Hard v0.1 (Li T. et al., 2024). Their method integrates manual curation, semi-supervised clustering, and stratified sampling to create balanced evaluation datasets covering diverse domains such as law, medicine, and multilingual contexts. This approach significantly improves benchmark separability (84%) and agreement with human preferences (84%), demonstrating the importance of tailored evaluation datasets for LLM-as-a-Judge frameworks. Our study aligns with this research by constructing manually labeled validation sets for search query parsing evaluation, ensuring that assessments align with human preferences and domain-specific accuracy requirements.

Beyond domain-specific benchmarks, LLM-as-a-Judge has also been explored in reasoning-intensive retrieval tasks. JudgeRank (Niu et al., 2024) introduces a three-step agentic reranking approach, where query analysis, document summarization, and relevance judgment are performed sequentially to improve retrieval accuracy in retrieval-augmented generation (RAG) systems. Their method outperforms dense retrieval baselines on reasoning-intensive tasks, highlighting the potential of LLMs in structured evaluation. While JudgeRank focuses on ranking search results, its stepwise reasoning approach informs our multi-step query parsing evaluation framework, where LLMs assess query understanding based on extracted structured attributes rather than document rankings.

Finally, evaluating semantic parsing for knowledge-based conversational question answering has revealed important insights into LLM performance on structured outputs. Schneider et al. (2024) evaluate LLMs in generating structured graph queries from natural language, demonstrating that few-shot prompting and fine-tuning techniques improve performance on structured parsing tasks. Their findings suggest that zero-shot performance is often inadequate for complex structured outputs, reinforcing our decision to incorporate few-shot prompting and iterative refinements in LLM-based search query parsing evaluation.

Overall, these studies highlight the importance of domain-specific criteria, specialized benchmarks, reasoning-driven evaluation strategies, and structured query assessment in adapting LLM-as-a-Judge to search query parsing and semantic parsing applications. Our work extends these efforts by introducing a scalable, structured evaluation pipeline, leveraging LLM-as-a-Judge for assessing query parsing outputs in real-world search systems.

## 3 Methodology

In this section, we propose a general framework for evaluating structured outputs using the LLM-as-a-Judge approach. Structured outputs, such as those in semantic and search query parsing, require both semantic understanding and structural consistency, making their evaluation more complex than rule-based assessments. We demonstrate our framework through the evaluation of search query parsing in an online advertisement platform. However, the proposed approach is not limited to this task. Instead, it provides a scalable and adaptable evaluation methodology for assessing structured outputs across various domains. To ensure reliable and interpretable evaluations, our framework incorporates structured evaluation prompts. Additionally, we introduce a Contextual Evaluation Prompt Routing strategy to improve evaluation efficiency and mitigate hallucinations in LLM-based assessments.

**Figure 2:**
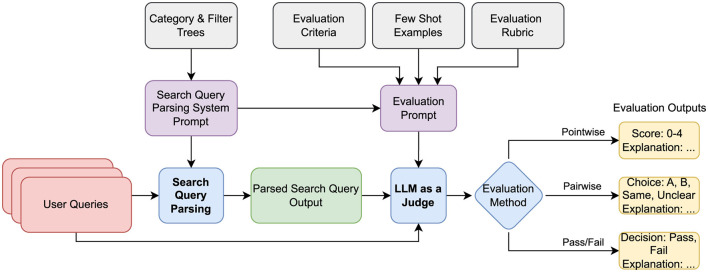
LLM-as-a-Judge framework.

The LLM-as-a-Judge framework proposed in this work is depicted in Figure 2. The framework follows a structured approach to assess the quality of the results of the parsed search query. The process begins with a user query that is given to the search query parser. The search query parser converts the query into a structured format, capturing attributes such as category, filters, location, and keywords by making use of information about categories (vehicle, real estate, etc.) and filters (vehicle condition, room size, etc.) encoded in trees. This parsed output is then evaluated through three distinct methods: pointwise evaluation, pairwise evaluation, and pass/fail evaluation. To perform evaluations, an evaluation prompt is given to the LLM-as-a-Judge as input. This evaluation prompt consists of domain-specific evaluation criteria, rating rubrics customized for each evaluation method, enough few-shot examples so that the LLM can learn how to evaluate from context, and the search query parsing system prompt to understand the category and filter information. Each evaluation method ensures transparency by providing a justification for its evaluation, reducing the reliance on human intervention while maintaining high reliability. By leveraging these techniques, the LLM-as-a-Judge framework offers a robust and scalable solution for evaluating structured outputs across diverse applications.

### 3.1 Search query parsing system

In this study, we evaluate a search query parsing system designed for an online classified advertisement platform. The parsing system plays a crucial role in understanding user queries and transforming them into a structured representation that enhances search accuracy and filtering capabilities. In this section, we describe briefly the query parsing system used in this work to understand the evaluation process clearly.

#### 3.1.1 Query types and user intent

The platform handles a diverse set of user search queries, ranging from simple keyword-based searches to more complex, multi-faceted queries that include specific filters and conditions. The queries typically fall into the following categories:

- Generic queries: broad search terms without specific filters (e.g., “cars for sale,” “rental apartments”).
- Feature-specific queries: queries that include attributes such as price range, brand, or room count (e.g., “red BMW under 500,000 TL,” “2 + 1 apartments for rent in Beşiktaş”).
- Location-based queries: queries that explicitly mention cities, districts, or neighborhoods (e.g., “houses for sale in Kadıköy,” “office space for rent in Levent”).
- Implicit intent queries: queries where certain attributes (e.g., price expectations such as “bargain price,” conditions like “urgent sale”) are implied rather than explicitly stated.

To ensure an optimal search experience, the search query parsing system must effectively interpret these queries, extract meaningful attributes, and represent them in a structured format.

#### 3.1.2 Structured output representation

The parsing system converts each query into a structured JSON output, ensuring that the search criteria in the query are properly categorized and formatted for the retrieval engine. All categories and filters used to extract structured output in this system are specific to the online classified platform domain. This structured output consists of the following key components:

- The search query parsing system classifies each query into a multi-level category hierarchy, ensuring that the search intent is accurately captured and aligned with the platform's structured taxonomy. At the highest level, category_level_1 represents the broad category, such as real estate. This is further refined into category_level_2, which indicates the general classification within the broader category, such as apartments or commercial properties. category_level_3 provides a more specific classification, identifying distinctions like for sale or for rent. In some cases, an additional level, category_level_4, is included for further refinements. Accurately determining the category of a query is essential, as it ensures that relevant filters and retrieval mechanisms are applied correctly, improving the precision of search results.
- The system also performs filter extraction, identifying both explicit and implicit filters within the query. Filters capture essential attributes that refine the search results and enhance user experience. These include numerical filters such as price ranges, mileage, and room counts (e.g., “houses under 5 million TL”); boolean filters which indicate conditions (e.g., “furnished” or “new”); and enumerated filters which define specific values such as brand names, fuel types, or transmission types. For reliable query interpretation, the extracted filters must be mapped accurately to the predefined system filters, ensuring structured and meaningful retrieval.
- The system also extracts location information, if present in the query. Location data is mapped to structured fields such as city (e.g., “Istanbul”) and district (e.g., “Kadıköy”). If the query lacks explicit location details, the system may infer relevant location attributes based on user behavior, default preferences, or additional context. Proper location extraction ensures that the results are relevant to the user's intent and geographical constraints.
- Keyword and synonym recognition plays a crucial role in enhancing search coverage and query understanding. The system identifies explicit keywords that appear in the user query, while also generating synonyms to improve search recall (e.g., “flat” for “apartment,” “auto” for “car”). However, if a keyword is already categorized under the category or filter fields, it is not duplicated as an explicit keyword to avoid redundancy. This structured approach to keyword and synonym recognition helps refine search accuracy while maintaining query clarity.

By converting unstructured natural language queries into structured data, the search query parsing system enhances the efficiency of the search engine. However, ensuring the accuracy of these parsed outputs requires a robust evaluation framework. This is where the LLM-as-a-Judge evaluation methodology is applied, assessing the correctness of structured outputs using various evaluation techniques described in the following section.

### 3.2 Evaluation methods

To systematically assess the quality of structured search query parsing outputs, we employ the LLM-as-a-Judge framework with three distinct evaluation methods: Pointwise, Pairwise, and Pass/Fail evaluation. Each of these methods leverages predefined evaluation criteria and structured rating rubrics to ensure consistency, transparency, and alignment with human assessments.

For all evaluation methods, we use the evaluation criteria outlined in Table 1 which ensure that the key aspects—category accuracy, filter accuracy, location accuracy, keyword accuracy, synonym accuracy, and completeness—are systematically assessed. Furthermore, each evaluation prompt incorporates few-shot examples to provide the LLM with contextual understanding and enable it to generate well-grounded assessments.

**Table 1:** Evaluation criteria for search query parsing.

**Evaluation criteria**	**Description**
Category accuracy	Ensures that the assigned category levels (1–4) are consistent with the category hierarchy. Level 4 can be set to “None” but must be accurate if present
Filter accuracy	Ensures the accuracy and completeness of extracted filters, including numerical ranges and boolean flags. Filters must be explicitly stated or strongly implied in the query and must adhere to the provided filter tree
Location accuracy	Checks whether location extraction is accurate, ensuring that the field is left as “None” if location details are absent or ambiguous in the query
Keyword accuracy	Assesses whether explicit and implicit keywords are correctly identified. Keywords that match a category name or filter should not be counted as explicit keywords. Implicit keywords should be judged in context but should not be penalized if absent
Synonym accuracy	Evaluates the correctness and relevance of synonyms. There should be no more than one synonym per keyword. If synonyms are absent, it should not be penalized. Synonyms should only be provided when they improve search clarity. Minor inaccuracies in synonyms should not be penalized
Completeness	Ensures that the JSON response contains all required fields (categories, filters, keywords, synonyms, and location where applicable). “None” values are allowed if the information is not in the query

The general evaluation pipeline is as follows:

1. Query parsing: a user query is parsed into a structured JSON format as explained in Section 3.1.2.
2. Evaluation prompt construction: an evaluation prompt is generated, incorporating the user query, the parsed output, the evaluation criteria, the rating rubrics, and few-shot examples.
3. LLM-based assessment: the LLM-as-a-Judge evaluates the parsed output using the selected evaluation method. The LLM assigns a score (for Pointwise Evaluation), selects a preferred response (for Pairwise Evaluation), or classifies the response as pass/fail (for Pass/Fail Evaluation), accompanied by an explanation.
4. Consistency: each evaluation is repeated multiple times with different runs. The average value for Pointwise Evaluation and the majority voting value for the other two evaluation methods is used to obtain reliable assessments.

#### 3.2.1 Use of few-shot examples in evaluation prompts

To enhance the reliability and reasoning capabilities of the LLM-as-a-Judge framework, we iteratively constructed and incorporated few-shot examples into the evaluation prompts. These few-shot examples are entirely independent of the validation dataset and were manually crafted to reflect a diverse range of user queries and parsing scenarios.

Each few-shot example consists of a user query, a corresponding structured output (either fully correct, partially correct, or incorrect), a detailed evaluation of that output based on the established evaluation criteria, and a final judgment (score, preference, or pass/fail decision) with justification. This structure provides the LLM with contextual grounding and helps calibrate its evaluation behavior across diverse parsing outcomes.

Experiments were conducted using both zero-shot and few-shot versions of the evaluation prompts. Initially, evaluation was performed using zero-shot prompts to identify common failure patterns. These insights were then used to iteratively design targeted few-shot examples that address specific weaknesses observed in the model's judgments. This adaptive refinement continued across multiple iterations, with new few-shot cases added in response to the evaluator errors, particularly focusing on challenging or ambiguous cases. Separate sets of few-shot examples were maintained and updated for each evaluation method (Pointwise, Pairwise, and Pass/Fail), ensuring that the prompting remained method-specific and aligned with the underlying rating rubrics.

The number of few-shot examples varied by evaluation type and category but generally started with around five examples per prompt. As iterations progressed and the evaluator's weaknesses became better understood, the number of few-shot examples increased, culminating in ~30 examples in the final prompt configurations. This iterative augmentation process significantly improved evaluator consistency and performance, as evidenced by the quantitative results reported in Section 4.4.

#### 3.2.2 Pointwise evaluation

**Figure 3:**
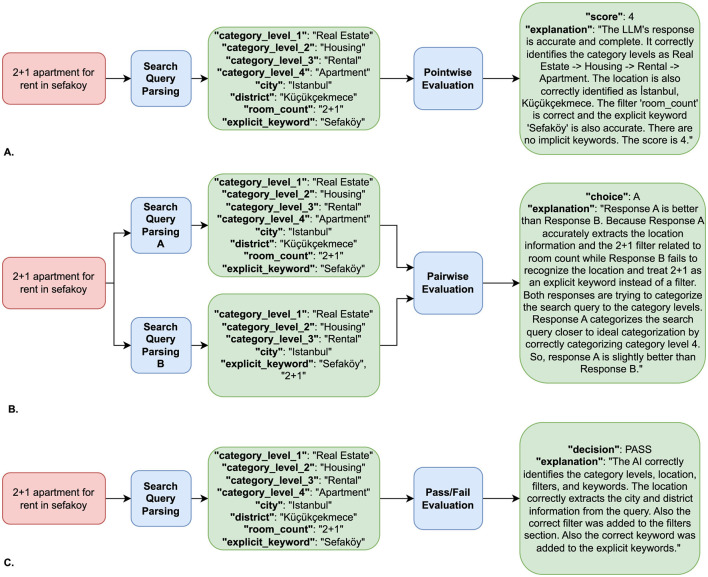
Running examples for each evaluation method. **(A)** Pointwise evaluation for an example query. **(B)** Pairwise evaluation for an example query. **(C)** PassFail evaluation for an example query.

Pointwise evaluation assesses the parsed query outputs by assigning a numerical score based on the predefined evaluation criteria and rating rubrics. The rating rubric we used for this method is shown in Table 2. The LLM evaluates the correctness of the parsed query with respect to the user query, assigns a score on a Likert scale (0–4), and also provides a textual justification for its rating. A running example of the pointwise evaluation process is illustrated in Figure 3A. An example evaluation prompt used in the pointwise evaluation can be found in Supplementary material.

**Table 2:** Evaluation rating rubrics.

**Rating**	**Description**
**Pointwise evaluation rating rubric**	
4 (Excellent)	The JSON response is completely accurate and complete, correctly identifying all categories, filters, keywords (explicit and implicit), synonyms, and location information (if applicable). It perfectly reflects the user's search intent.
3 (Good)	The JSON response is mostly accurate and complete, with only minor inaccuracies or omissions.
2 (Fair)	Several inaccuracies or omissions impact the overall understanding of the user's search intent.
1 (Poor)	The JSON response is largely inaccurate and incomplete, failing to capture the essence of the query.
0 (Unacceptable)	The response is completely incorrect or empty.
**Pairwise evaluation rating rubric**	
A	Response A is significantly better than Response B.
B	Response B is significantly better than Response A.
SAME	Both responses are nearly identical in performance.
UNCLEAR	Neither response accurately reflects the user's search intent.
**Pass/fail evaluation rating rubric**	
PASS	The response meets all correctness criteria.
FAIL	The response contains substantial errors or omissions.

#### 3.2.3 Pairwise evaluation

Pairwise evaluation compares two different parsed outputs for the same query, enabling a direct comparison between different search query parsing models. This method may be particularly useful for assessing performance improvements between traditional rule-based systems and LLM-based systems. The LLM determines which output better meets the evaluation criteria or declares them equivalent if both are equally valid or unsatisfactory. The rating rubric used for pairwise evaluation is given in Table 2. Figure 3B shows an example that compares the outputs of two parsing systems for the same query. An example evaluation prompt used in pairwise evaluation can be found in Supplementary material.

#### 3.2.4 Pass/fail evaluation

The Pass/Fail evaluation simplifies the assessment by converting the evaluation process into a binary classification task. Instead of assigning scores or making comparative judgments, the LLM assesses whether the parsed output meets the evaluation criteria and classifies it as either “PASS” or “FAIL.” The rating rubric is given in Table 2. Figure 3C presents a sample Pass/Fail evaluation scenario. An example evaluation prompt used in Pass/Fail evaluation can be found in Supplementary material.

### 3.3 Contextual evaluation prompt routing

We propose a method called Contextual Evaluation Prompt Routing to improve both the reliability and efficiency of LLM-based evaluation in structured output tasks. This approach dynamically routes evaluation prompts based on the category of the user query, enabling the use of tailored evaluation criteria, category-specific rating rubrics, and customized few-shot examples aligned with domain-specific parsing expectations.

Prior to this approach, we used a single unified evaluation prompt for all query categories as detailed in Section 3.2. The prompt included a comprehensive set of evaluation rubrics, criteria, and few-shot examples covering all categories. However, experimental analysis revealed that this one-size-fits-all design introduces several key limitations:

- First, criteria that are important in one domain (e.g., Location Accuracy in real estate category) are not that much important in other domains, leading the LLM evaluator to misinterpret irrelevant or inapplicable instructions.
- Second, the inclusion of few-shot examples from unrelated domains increases hallucination risk, as the model might align the evaluation with incorrect reference structures.
- Third, as we expand the number of few-shot examples to improve performance, the token length of the prompts exceeds practical limits (up to 100k tokens), resulting in degraded performance and higher computational cost.

To mitigate these issues, the Contextual Evaluation Prompt Routing strategy segments the evaluation process into two stages. First, the structured output's category_level_1 value is used to determine the main category of the query. Then, a category-specific evaluation prompt is constructed with

- Only the relevant evaluation criteria and rating rubric,
- Tailored few-shot examples that reflect the annotation standards of that specific category, and
- A more compact and focused prompt length, improving LLM interpretability.

**Figure 4:**
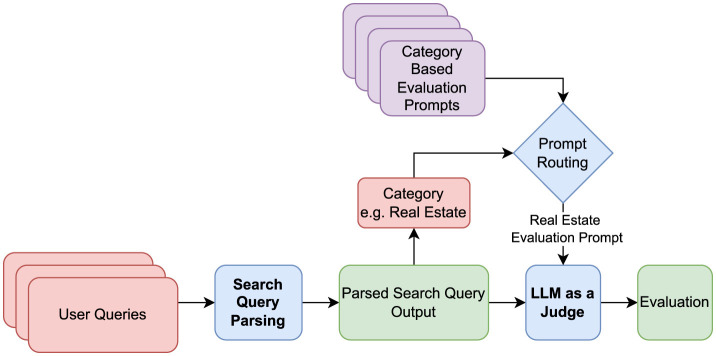
Contextual evaluation prompt routing pipeline.

This strategy offers several advantages. By eliminating irrelevant instructions and examples, it reduces hallucinations and improves evaluation accuracy through domain-specific guidance. The modular nature of the prompts also significantly lowers the inference time and computational costs due to shorter input lengths. Moreover, isolating category-specific configurations allows for the inclusion of a larger number of relevant few-shot examples, further enhancing the evaluation performance. The overview of the routing mechanism is depicted in Figure 4, and a sample routed prompt is provided in Supplementary material.

The proposed method is conceptually inspired by advances in task decomposition and prompt specialization. For instance, Multi-Trait Specialization (MTS) (Lee et al., 2024) applies trait-specific prompts to improve zero-shot performance in essay scoring. While MTS addresses unstructured generation tasks, our Contextual Evaluation Prompt Routing strategy adapts the underlying principles to the structured output domain of semantic search query parsing. Khot et al. (2023) introduce decomposed prompting that modularizes complex tasks into simpler, interpretable components. Dun et al. (2025) propose the Mixture of Prompts (MoPs) framework, which dynamically selects specialized prompt modules based on input characteristics, improving adaptability across heterogeneous tasks. Building on these insights, our routing method offers a scalable, interpretable, and efficient alternative to monolithic prompts for evaluation in multi-domain structured output settings.

While related to prior prompt specialization approaches, the proposed method is uniquely designed for structured evaluation scenarios that require strict rubric alignment, interpretability, and category consistency. Thus, Contextual Evaluation Prompt Routing is not just a heuristic routing solution, it also serves as a foundational mechanism for scalable and reliable deployment of LLM-as-a-Judge systems in real-world, multi-domain search applications.

To validate its effectiveness, we constructed category-specific evaluation subsets, each annotated by domain experts. These controlled benchmarks revealed that category-specialized routing improves both alignment with human judgments and consistency across repeated assessments. Importantly, the strategy generalizes well to unseen inputs without requiring model fine-tuning, supporting its modularity and adaptability to diverse semantic parsing domains. Furthermore, we applied a range of statistical methods, including agreement metrics and error analysis, to quantify the reliability and stability of our evaluation framework. The results indicate that using category-specialized prompts not only improves alignment with human assessments but also enhances evaluation robustness without introducing the cost overhead associated with full-scale model fine-tuning.

Through the methodology explained in Sections 3.2 and 3.3, the proposed LLM-as-a-Judge framework offers a modular, scalable, and semantically accurate evaluation solution for structured search query parsing. By integrating multiple evaluation strategies and enhancing them with domain-aware prompt routing, our approach achieves high interpretability and consistency while significantly reducing the need for human evaluation in critical systems.

## 4 Experiments and results

In this section, we present the datasets, the evaluation metrics, and the experimental setup used for assessing the effectiveness of the LLM-as-a-Judge framework in evaluating search query parsing systems. Our goal is to systematically compare different evaluation methods (Pointwise, Pairwise, and Pass/Fail) and evaluation techniques (in-context learning, prompt engineering with iterative refinement, human-in-the-loop evaluation, etc.) and validate their reliability in capturing the accuracy and completeness of structured query parsing outputs. In addition, we investigate the impact of the Contextual Evaluation Prompt Routing strategy on the consistency and efficiency of the evaluation. The results are presented accompanied with ablation studies. We also conduct an error analysis and discuss the limitations of the proposed approach.

### 4.1 Datasets

In this work, we compiled two datasets and conducted the experiments on these datasets.^1^

#### 4.1.1 Small-scale evaluation dataset

The first dataset consists of 66 queries covering various search scenarios. These queries were manually created by domain experts and designed to represent different category levels, filtering attributes, and query complexities commonly encountered in search systems. Each query was processed by two different search query parsers:

- Rule-based parser: a traditional query parsing system that relies on predefined rules and heuristics to extract structured information.
- LLM-assisted parser: a more flexible, context-based parser that leverages a large language model to interpret and generate structured query representations.

For each query, the structured outputs generated by both parsers are manually evaluated and labeled by a human annotator with domain knowledge and background in computer science. The annotator assigns scores to the search query parser outputs for Pointwise evaluations, makes pass/fail decisions for Pass/Fail evaluations, and determines which of the two parsing outputs better captures the search intent for Pairwise evaluations. To address the known position bias issues in Pairwise evaluation, in half of the cases the first parser output and in the other half of the cases the second parser output are presented first to the LLM evaluator. The annotations are then reviewed by five domain experts who provide feedback and additional notes to ensure accuracy and consistency. Based on this expert feedback, the annotator revisits and refines the labels, ensuring that the final annotations align with domain-specific expectations and serve as reliable ground-truth references.

#### 4.1.2 Large-scale evaluation dataset

To further analyze the performance of the LLM-as-a-Judge framework at scale, we constructed a more extensive dataset consisting of 600 queries spanning multiple domain-specific categories. These queries were selected according to search frequency after removing duplicate and highly similar queries among the most frequently searched queries on the online classified platform. These categories align with the main categories used in the routing strategy and include “Real Estate,” “Vehicles,” “Used & Brand New Items,” “Vehicle Parts, Accessories & Tuning,” “Other Categories,” and “No Category” (Uncategorized Queries) categories.

One hundred queries were collected for each category, ensuring a balanced representation of search intents and parsing challenges. Given the high annotation cost of the large-scale dataset and based on the experimental findings in the small-scale dataset that show that Pass/Fail evaluation yields more consistent results, the large-scale dataset was only annotated for the outputs of the LLM-based parser for Pass/Fail evaluation. The annotation process followed a similar labeling procedure as the small-scale dataset. However, to accelerate the manual annotation process, a preliminary annotation was first conducted using the best-performing techniques identified in the small-scale dataset. The outputs were then manually reviewed and refined by human annotators to ensure high-quality ground truth labels.

This larger dataset enabled a more comprehensive assessment of the Contextual Evaluation Prompt Routing strategy, allowing us to evaluate how well domain-specific prompts improve the accuracy and reliability of search query parsing evaluations across different query characteristics, such as query length, complexity, and category-specific constraints.

### 4.2 Evaluation metrics

To assess the effectiveness of the LLM-as-a-Judge framework, we employed a range of evaluation metrics that measure the alignment between LLM-based evaluations and human judgments. We selected a number of proper metrics for each evaluation method. The metrics were used to quantify both the agreement with human evaluations and the reliability of the LLM-based assessment process.

#### 4.2.1 Agreement metrics

We utilized different agreement metrics depending on the evaluation methodology.

##### 4.2.1.1 Pointwise evaluation

Since Pointwise evaluation involves assigning numerical scores to structured query parsing outputs, we used correlation metrics below to measure the alignment between LLM-generated scores and human ratings:

- *Spearman's rank correlation (*ρ*)* was used to assess the monotonic relationship between the rankings of human and LLM evaluations. This metric evaluates whether higher scores assigned by humans correspond to higher scores assigned by the LLM.
- *Standard deviation across runs* reflects the average variability of scores assigned by the LLM across evaluation runs per query, indicating the consistency of the LLM-as-a-Judge system for a given setup.

##### 4.2.1.2 Pairwise and pass/fail evaluation

Since these evaluation methods involve categorical decisions rather than numerical scores, we used classification-based agreement metrics:

- *Exact match accuracy* was used to measure the percentage of instances where the LLM's decision matched the human annotated label. This metric is a simple but effective way to calculate the agreement of categorical evaluations.
- *Cohen's Kappa (*κ*)* was utilized to account for agreement beyond chance, measuring the level of consistency between human and LLM evaluations while considering the possibility of random agreement. This metric is particularly useful for categorical classification.

#### 4.2.2 Reliability metrics

In addition to aligning with human annotations, we assessed the internal reliability of the LLM evaluator using statistical measures that quantify the consistency of its evaluations across different subsets. Unlike conventional inter-rater agreement metrics, which evaluate the consensus among different raters, our approach measures the variability of LLM judgments using standard deviation, coefficient of variation, and mean absolute deviation. These metrics provide a robust assessment of how consistently the LLM applies its evaluation criteria across diverse query distributions.

The standard deviation (SD) measures how much the agreement scores deviate from their mean. A lower standard deviation indicates that the evaluations are consistent and stable, while a higher value suggests fluctuations in agreement scores, pointing to inconsistencies in LLM assessments. This metric is useful for understanding the overall dispersion of scores across different evaluation runs.

The coefficient of variation (CV) normalizes the standard deviation by expressing it as a percentage of the mean, making it useful for comparing variability across different datasets. A lower CV percentage suggests more stable evaluations, while a higher percentage indicates greater variability. Since CV accounts for differences in scale, it helps in making meaningful comparisons between categories with varying levels of agreement scores.

The mean absolute deviation (MAD) measures the average absolute difference between the agreement scores and their mean. Unlike standard deviation, MAD is less sensitive to extreme outliers, making it a robust alternative for measuring variability. A lower MAD value suggests that evaluations remain closely distributed around the mean, while a higher value indicates larger fluctuations and potential inconsistency in LLM-based judgments.

This combination of agreement and reliability metrics provides a comprehensive assessment of the LLM-as-a-Judge framework, ensuring that the evaluation process is both aligned with human judgments and reliable.

### 4.3 Experimental setup

In the experiments, we evaluated the LLM-as-a-Judge framework using a range of Gemini^2^ models: gemini-1.5-flash-001, gemini-1.5-flash-002, gemini-1.5-pro-001, and gemini-1.5-pro-002. The flash models are optimized for efficiency, being smaller and faster than the pro models. Specifically, gemini-1.5-flash-002 represents an update over 001. We initially began with Pointwise evaluation, which is relatively more complex than the other evaluation methods. For this task, we primarily employed the pro models due to their higher capacity, and ran each prompt setup multiple times to ensure the stability of the results. Given the high computational cost of the pro models, we continued with the more cost-efficient flash models in the later stages. Additionally, as new Gemini models were released during the study, we updated the models used in our experiments accordingly. As a result, we used the most suitable models in different stages of the experiments based on the complexity of the evaluation and the cost considerations. By comparing these models, we aimed to understand the impact of model size, optimization strategies, and generational advancements on the framework's performance.

We configure the temperature and seed parameters during inference to ensure controlled and reproducible evaluations. The temperature is set to 0.7, allowing for a balanced degree of randomness in token selection while maintaining response consistency. The seed parameter is specified to enforce deterministic outputs. However, due to the probabilistic nature of LLMs, setting a fixed seed does not entirely eliminate the variance in responses across multiple runs.

Although LLM evaluation is inherently a deterministic task, variability in token selection may lead to slight inconsistencies in the generated evaluations. We perform multiple evaluation iterations for each experimental setup to mitigate this issue. By running multiple iterations, we ensure the stability of LLM evaluations and reduce the impact of randomness on the assessment metrics. This iterative approach helps quantify the robustness of the LLM evaluators across different evaluation methodologies.

### 4.4 Results

This section presents the results of the experiments for the LLM evaluation of search query parsing outputs. We analyze the impact of various evaluation methods, prompt designs, evaluator models, and reference values on alignment with human judgments and evaluation consistency.

As stated in Section 4.1, the queries in the small-scale dataset were parsed by both the rule-based parser and the LLM-based parser, and the results of both parsers were manually annotated. We thus evaluate the outputs of both parsers using the LLM-as-a-Judge framework for the small-scale dataset and compute the agreement scores with the human labels. For Pointwise and Pass/Fail evaluations the parsed outputs of the two parsers are assessed separately, while for Pairwise evaluation the two parsers are compared in the same experiment. Since the Contextual Evaluation Prompt Routing strategy was tested across all three evaluation methodologies (Pointwise, Pairwise, and Pass/Fail), the experiments leveraging this strategy were conducted using the small-scale validation dataset where both parsers' outputs were annotated. Due to the annotation cost, the large-scale dataset was only used to evaluate the effectiveness of the strategy in the Pass/Fail setting, where only the LLM-based parser's outputs were labeled.

#### 4.4.1 Pointwise evaluation results

Tables 3, 4 show the evaluation results of the parsed queries for the two parsers. In all the tables in this section, we include the results only for the gemini-1.5-flash-002 and gemini-1.5-pro-002 models which are newer versions of the flash and pro models in order not to clutter the tables. The complete results for all the models and configurations are provided in Supplementary material. The LLM evaluation for each query and setting is repeated 10 times and averaged to increase the reliability of the scores. The Spearman's correlation column is the correlation between the human scores and the LLM scores (averaged). In addition to Spearman's correlation, Pearson and Kendall's Tau correlation metrics were also computed. However, as their results were consistent with the Spearman correlation values and did not provide additional insights, we do not include them in the tables. Table 5 shows the interpretation of the Spearman's correlation values. The standard deviation column is the standard deviation of the 10 runs. Having multiple runs helps account for the variability in the LLM outputs, ensuring that our evaluation captures consistency across different test cases.

**Table 3:** Pointwise evaluation results for LLM-based parser outputs.

**Evaluator model**	**Prompt type**	**Spearman's correlation**	**Standard deviation across runs**
gemini-1.5-flash-002	Basic prompt	0.381	0.023
	Basic prompt + few shot	0.364	0.023
	Basic prompt + few shot + Explain first	0.350	0.184
	Basic prompt + few shot + Separate system prompt	0.565	0.020
	Basic prompt + few shot + Reference values	0.898	0.024
	Prompt routing	0.402	0.092
	Prompt routing + few shot (initial)	0.664	0.081
gemini-1.5-pro-002	Basic prompt	0.490	0.061
	Basic prompt + few shot	0.445	0.464
	Basic prompt + few shot + explain first	0.461	0.453
	Basic prompt + few shot + separate system prompt	0.354	0.402
	Basic prompt + few shot + reference values	0.858	0.209
	Prompt routing	0.481	0.181
	Prompt routing + few shot (initial)	0.671	0.151

**Table 4:** Pointwise evaluation results for rule-based parser outputs.

**Evaluator model**	**Prompt type**	**Spearman's correlation**	**Standard deviation across runs**
gemini-1.5-flash-002	Basic prompt	0.564	0.082
	Basic prompt + few shot	0.793	0.057
	Basic prompt + few shot + separate system prompt	0.785	0.015
	Basic prompt + few shot + reference values	0.870	0.007
	Prompt routing	0.371	0.127
	Prompt routing + few shot (initial)	0.309	0.102
gemini-1.5-pro-002	Basic prompt	0.677	0.062
	Basic prompt + few shot	0.853	0.508
	Basic prompt + few shot + separate system prompt	0.711	0.458
	Basic prompt + few shot + reference values	0.832	0.492
	Prompt routing	0.802	0.497
	Prompt routing + few shot (initial)	0.815	0.186

**Table 5:** Interpretation of Spearman's rank correlation (adapted from Dancey and Reidy, 2007).

**Spearman's rank correlation**	**Interpretation**
≥0.70	Very strong relationship
0.40–0.69	Strong relationship
0.30–0.39	Moderate relationship
0.20-0.29	Weak relationship
0.01–0.19	No or negligible relationship

*Basic prompt* denotes the prompt in its basic form and is shown in Supplementary material. *Few-shot* shows the effect of including query examples in the prompt (Section 3.2.1) to guide the LLM evaluation. While evaluating a parser output, the LLM gives a score which is followed by an explanation. *Explain first* shows the results when the LLM is guided to explain the score before presenting the score, which was observed to increase the evaluation performance in some works. *Separate system prompt* refers to a prompt design in which the search query parser's original system prompt and the user query are presented in separate sections, rather than being concatenated and given as a single input. This aims to make the evaluation criteria clearer by structurally distinguishing between system behavior and user input. In order to apply the separate system prompt, the user query should be removed from the Search Query Parser Prompt section in the evaluation prompt and written under a separate heading. *Reference values* indicates whether the gold (human-annotated) reference parsing output is provided within the evaluation prompt. When reference values are included, the LLM-as-a-Judge can see what the correct structured output should look like, allowing it to make more informed and accurate evaluations.

It is important to note that there is a significant score gap between the evaluation of the rule-based and LLM-based parser outputs. This discrepancy primarily stems from the nature of the rule-based parser, which either parses a query very well or very poorly due to extensive manual mappings and highly domain-specific rules. As a result, the LLM evaluator often assigns either the highest or lowest score, which simplifies its decision-making and leads to higher agreement with human ratings. In contrast, the LLM-based parser tends to produce more nuanced outputs with partial correctness. In these borderline cases, the LLM evaluator may either overlook minor errors and assign a high score or interpret them as critical issues and give a low score, both of which hinder alignment with human judgment.

##### 4.4.1.1 Impact of adding few-shot examples

Tables 3, 4 reveal different impacts of few-shot prompting across the two types of parser. For the LLM-based parser, adding few-shot examples does not improve alignment with human scores; in fact, a slight drop is observed. For example, in gemini-1.5-flash-002, Spearman's correlation decreases from 0.381 to 0.364, and in gemini-1.5-pro-002 from 0.490 to 0.445. This decline can be attributed to the fact that the LLM-based parser already produces semantically nuanced outputs. Introducing few-shot examples may have led the LLM evaluator to overfit to specific reference patterns in the prompt, resulting in misalignment for more ambiguous or borderline cases.

In contrast, for the rule-based parser, few-shot prompting yields substantial improvements. Correlation increases from 0.564 to 0.793 in gemini-1.5-flash-002 and from 0.677 to 0.853 in gemini-1.5-pro-002, representing a clear boost in evaluation accuracy. This effect is likely due to the more rigid and deterministic nature of rule-based outputs, which align better with the explicit decision templates presented in few-shot examples. The examples provide clear guidance that helps the LLM evaluator assess structured, rule-derived outputs more effectively.

##### 4.4.1.2 Impact of contextual evaluation prompt routing

The addition of Contextual Evaluation Prompt Routing yields different effects depending on the parser type. For the LLM-based parser, routing alone results in a modest improvement or similar performance over the basic prompt (0.402 and 0.481 Spearman correlation for flash-002 and pro-002, respectively). When combined with category-specific few-shot examples, the alignment further improves to 0.664 and 0.671 for the two models, respectively. This reflects a transition from moderate to strong correlation (Table 5), suggesting that contextual prompt routing helps the LLM evaluator better understand structured outputs through more targeted domain-specific guidance.

For the rule-based parser, however, the effect is more nuanced. In the flash-002 model, routing alone or with few-shot examples does not improve performance and in fact leads to a decline (0.371 and 0.309 vs. 0.564 with the basic prompt). This may be due to the simpler and highly rigid outputs of the rule-based system, which align better with generic evaluation instructions rather than segmented category-specific ones. However, in the pro-002 model, prompt routing continues to be beneficial, achieving 0.802 correlation without few-shot and 0.815 with category-specific few-shot examples, both signaling improvement over the basic prompt (0.677). These results suggest that routing is more effective when combined with stronger evaluator models that can make use of nuanced prompt variations.

Overall, the findings indicate that Contextual Evaluation Prompt Routing, especially when paired with few-shot examples, is a promising approach for increasingrere alignment in LLM-based evaluations, particularly for LLM-based parser outputs.

##### 4.4.1.3 Impact of evaluator model

The choice of the evaluator model significantly affects the reliability and quality of LLM-based evaluations. Across our experiments, we observed that different sizes of the Gemini family models (flash-002 and pro-002) demonstrate varying levels of correlation with human judgments and stability across runs, even under identical prompting conditions.

Interestingly, the smaller model gemini-1.5-flash-002 achieves the highest Spearman correlation among all configurations for LLM-based parser outputs when reference values are provided (0.898), outperforming the larger pro-002 model (0.858). Similarly, in the rule-based parser evaluation, flash-002 reaches a peak correlation of 0.870, slightly higher than pro-002 (0.832). These results suggest that smaller models can be more effective than larger ones in alignment with human ratings when given strong reference cues.

However, flash-002 also shows more pronounced variability across different prompting strategies. For example, in the LLM-based parser, Spearman correlation drops to 0.350 with the Explain First prompt, compared to 0.565 with Separate System Prompt, indicating greater sensitivity to prompt format. On the other hand, pro-002 tends to offer more stable performance across configurations, with smaller fluctuations in correlation values between prompt types.

Standard deviation results further support this observation. Under basic prompt conditions, pro-002 exhibits low variability (0.061 and 0.062 for LLM-based and rule-based parsers), whereas flash-002 shows very low variability only in its simplest configurations (0.023) but higher variance in others (e.g., 0.184 in Explain First). This implies that while both models can reach strong alignment with humans under optimal prompting, the pro-002 model tends to produce more consistent evaluations across prompt types.

One notable and consistent observation is that individual evaluator models behave similarly when scoring both the LLM-based and rule-based parser outputs. That is, if a model performs well in evaluating the rule-based system, it tends to also perform well in the LLM-based system, under the same prompt configuration. This suggests that model behavior is influenced more by the evaluator's internal alignment mechanisms than by the type of the parser being evaluated.

Prompt length is another critical factor. Adding more few-shot examples or reference values generally increases prompt complexity, which can in turn lead to higher output variance. For instance, standard deviation reaches 0.464 with few-shot prompts in pro-002 (LLM-based parser), suggesting that model outputs become less stable when overwhelmed with too much contextual information.

Notably, prompt routing appears effective in reducing this variance. In the LLM-based parser evaluations, using prompt routing combined with few-shot examples yields standard deviations of 0.081 (flash-002) and 0.151 (pro-002), substantially lower than in their respective monolithic few-shot setups. These findings validate our hypothesis that modular, category-specific routing mitigates prompt overload and improves evaluation stability, especially when tailored few-shot examples are supplied for each domain.

Overall, these results highlight that while larger models like pro-002 may offer more predictable performance across settings, smaller models like flash-002 can deliver superior alignment when supported by strong reference guidance. Ultimately, careful balancing of model size, prompt strategy, and task-specific routing is key to achieving both accurate and consistent LLM-as-a-Judge evaluations.

##### 4.4.1.4 Impact of reference values

Providing reference values within the prompt mostly enhances alignment with human evaluations. In the LLM-assisted parser evaluations, the correlation improved from 0.445 to 0.858, moving from a strong relationship to a very strong relationship (Table 5), highlighting the importance of reference values in guiding the evaluation. Additionally, standard deviation values decreased from 0.464 to 0.209, reinforcing that reference values contribute to more stable evaluations.

##### 4.4.1.5 Impact of scoring first vs. explaining first

When the LLM is requested to provide a justification for the score before giving the score, the correlation with the human score increases. Table 3 shows that correlation increases from 0.445 to 0.461 for the stronger gemini-1.5-pro-002 model. The increase in correlation suggests that allowing the LLM to rationalize its decisions before scoring leads to more thoughtful and accurate evaluations. However, the variability in the evaluation runs does not follow a particular pattern.

After observing that the explain-first strategy yielded consistently better results than the score-first prompting for the LLM-based parser, all subsequent evaluations in this study were conducted using the explain-first strategy.

##### 4.4.1.6 Impact of providing system prompt separately

We observe that separating the system prompt from the user query, i.e. making the evaluation criteria distinct from the user input, results in lower Spearman correlation scores compared to other setups but yields more stable evaluations. This suggests that integrating the system prompt with the evaluation prompt provides additional context that aids assessment and keeping them separate improves consistency across multiple runs.

In particular, for rule-based parser outputs evaluated with gemini-1.5-pro-002, using the separated system prompt along with few-shot examples yielded a Spearman correlation of 0.711. This is lower than other configurations except the basic prompt, indicating that structurally separating prompt sections negatively impacts accurate evaluations.

#### 4.4.2 Pairwise evaluation results

Table 6 shows the evaluation results for Pairwise evaluation where we compare the outputs of the two parsers. As stated in Section 4.1.1, the dataset is randomly shuffled such that the output of the rule-based parser appears in the first position in half of the examples and the output of the LLM-based parser appears in the first position in the other half. The LLM evaluation for each query and setting is repeated 5 times and majority voting is applied. In *Few-Shot (Initial)*, we include 3 examples in the prompt. After the initial experiment with few-shot examples, we iteratively refine the prompt by analyzing the decisions of the LLM and adding new examples to the prompt according to the results of the analysis (Section 3.2.1). *Few-Shot (Final)* denotes the final form of the prompt in which 24 examples are used. We used the exact match and Cohen's Kappa to measure the alignment between human evaluations and LLM evaluations. Full evaluation results including experiments with gemini-1.5-pro-001 and gemini-1.5-flash-001 are provided in Supplementary material for completeness and reference.

**Table 6:** Pairwise evaluation results.

**Evaluator model**	**Prompt type**	**Exact match accuracy**	**Cohen's Kappa**
gemini-1.5-flash-002	Basic prompt	0.773	0.635
	Basic prompt + few shot (initial)	0.773	0.632
	Basic prompt + few shot (final)	0.879	0.807
	Prompt routing	0.697	0.508
	Prompt routing + few shot (initial)	0.758	0.607
gemini-1.5-pro-002	Basic prompt	0.758	0.610
	Basic prompt + few shot (initial)	0.758	0.610
	Basic prompt + few shot (final)	0.879	0.816
	Prompt routing	0.742	0.583
	Prompt routing + few shot (initial)	0.803	0.687

##### 4.4.2.1 Impact of few-shot prompting

Table 6 shows that, for the gemini-1.5-flash-002 model, using the basic prompt yields an exact match accuracy of 0.773 and a Cohen's Kappa of 0.635. While these results seem reasonable, they indicate room for improvement for the LLM evaluator in capturing nuanced differences between the system outputs. When a large number of examples is included in the prompt, the exact match accuracy is increased to 0.879 and the Cohen's Kappa to 0.807, demonstrating the effectiveness of few-shot learning in refining LLM-based evaluation. When switching from the gemini-1.5-flash-002 model to the more advanced gemini-1.5-pro-002 model using the same refined prompt, Cohen's Kappa increased slightly from 0.807 to 0.816, suggesting better agreement between the human and the LLM compared to random agreement.

These results confirm that adding more diverse few-shot examples enhances the evaluation accuracy, allowing the model to better differentiate between subtle variations in query parsing performance.

##### 4.4.2.2 Impact of contextual evaluation prompt routing

In addition to standard few-shot prompting, we evaluated the impact of Contextual Evaluation Prompt Routing in the pairwise setting. This strategy routes evaluation prompts based on the category of the search query to reduce prompt length and improve semantic relevance (see Section 3.3).

As shown in Table 6, prompt routing without few-shot examples results in lower performance than the basic prompt in both flash-002 and pro-002 models. For instance, exact match accuracy drops from 0.773 to 0.697 in flash-002, and Cohen's Kappa decreases from 0.635 to 0.508. This suggests that routing alone, without adequate in-context examples, may reduce the LLM's ability to generalize comparisons across domains.

However, when routing is combined with even a small number of few-shot examples [prompt routing + few shot (initial)], performance may improve substantially. For example, in pro-002, exact match accuracy increases to 0.803 and Cohen's Kappa to 0.687, outperforming the basic prompt setup. This confirms that routing and few-shot prompting are complementary in the sense that routing helps delivering category-relevant context while examples help guiding fine-grained decision boundaries in the pairwise comparison task.

These findings align with earlier trends observed in pointwise evaluations; routing by itself reduces prompt cluttering and improves stability, but gains are best realized when domain-specific few-shot guidance is also provided.

##### 4.4.2.3 Impact of position bias

Position bias is a well-known issue in pairwise evaluation, where models tend to favor responses in a particular position (first position or second position). To analyze the effect of position bias, we conducted an additional experiment using an unshuffled dataset. The output of one of the parsers always appears in the first position and the LLM is asked to select the better one among the two outputs. We used the gemini-1.5-flash-002 model and the refined prompt with large number of few-shot examples. Table 7 shows the results. Compared to the original setting where the order of the pairs are shuffled, the exact match accuracy decreased from 0.879 to 0.833 and Cohen's Kappa dropped from 0.807 to 0.639.

**Table 7:** The effect of position bias in pairwise evaluation.

**Dataset**	**Exact match accuracy**	**Cohen's Kappa**
Dataset with shuffled pairs	0.879	0.807
Dataset with no-shuffled pairs	0.833	0.639

This significant drop in inter-rater agreement highlights the importance of randomizing the response order in pairwise evaluation setups to prevent systematic biases. Without shuffling, the model may develop an unintended preference for responses in a specific position, leading to skewed evaluation results.

#### 4.4.3 Pass/fail evaluation results

Tables 8, 9 show the evaluation results for Pass/Fail evaluation for the outputs of the two parsers. The LLM evaluation for each query and setting is repeated 5 times and majority voting is applied. Similar to Pointwise evaluation, we started with a basic prompt without few shot examples and then used 15 examples in the initial few-shot setting. By analyzing the evaluations of the LLM, we refined the prompt to include more examples and used 30 examples in the final few-shot setting (Section 3.2.1). Full evaluation results including experiments with gemini-1.5-pro-001 and gemini-1.5-flash-001 are provided in Supplementary material for completeness and reference.

**Table 8:** Pass/Fail evaluation results for LLM-based parser outputs.

**Evaluator model**	**Prompt type**	**Exact match accuracy**	**Cohen's kappa**
gemini-1.5-flash-002	Basic prompt	0.742	0.282
	Basic prompt + few shot (initial)	0.727	0.250
	Basic prompt + few shot (final)	0.848	0.659
	Prompt routing	0.894	0.747
	Prompt routing + few shot (initial)	0.939	0.861
gemini-1.5-pro-002	Basic prompt	0.727	0.250
	Basic prompt + few shot (initial)	0.727	0.270
	Basic prompt + few shot (final)	0.848	0.625
	Prompt routing	0.818	0.526
	Prompt routing + few shot (initial)	0.909	0.780

**Table 9:** Pass/fail evaluation results for rule-based parser outputs.

**Evaluator model**	**Prompt type**	**Exact match accuracy**	**Cohen's kappa**
Gemini-1.5-flash-002	Basic prompt	0.485	0.040
	Basic prompt + few shot (initial)	0.894	0.787
	Basic prompt + few shot (final)	0.803	0.593
	Prompt routing	0.909	0.816
	Prompt routing + few shot (initial)	0.939	0.878
Gemini-1.5-pro-002	Basic prompt	0.848	0.692
	Basic prompt + few shot (initial)	0.909	0.815
	Basic prompt + few shot (final)	0.955	0.907
	Prompt routing	0.773	0.544
	Prompt routing + few shot (initial)	0.909	0.816

##### 4.4.3.1 Impact of few-shot prompting

Tables 8, 9 show that using a basic prompt without few-shot examples yields low agreement with human labels. When few-shot examples are included in the prompt, the agreement scores increase significantly for both parsers. Refining the prompt and adding more targeted examples further improves alignment with human decisions. We also tested whether increasing the number of examples beyond 30 leads to higher scores, but observed that prompt length issues began to reduce effectiveness. This aligns with prior findings indicating that overly long prompts may introduce confusion and hallucination in LLM-based evaluators (Zhang et al., 2024).

##### 4.4.3.2 Impact of fine-tuning the LLM evaluator

To address the prompt length and inference overhead challenges in few-shot prompting setups, we explored fine-tuning as an alternative strategy for enhancing the reliability of LLM-based evaluations in an additional experiment. Instead of incorporating few-shot examples directly into the prompt which may lead to prompt length constraints, we constructed a supervised dataset containing 57 manually curated examples, aggregated from the few-shot samples used in earlier prompting experiments. These examples span a range of query parsing outputs across multiple categories and were used to fine-tune the gemini-1.5-flash-002 model.

Although the fine-tuned models achieved improvements over the basic prompt setting by raising the exact match accuracy from 0.742 to 0.794 for the LLM-based parser and from 0.485 to 0.755 for the rule-based parser, they could not outperform the few-shot prompting configuration. Tables 8, 9 show that the final few-shot prompt setup achieved Exact Match Accuracy scores of 0.848 for the LLM-based parser and 0.803 for the rule-based parser in the gemini-1.5-flash-002 model, surpassing the fine-tuned model performance. This indicates that while fine-tuning provides a stable baseline improvement over naive prompting, few-shot prompting remains a more effective method, especially when sufficient in-context examples can be supplied.

To further explore this, we conducted additional experiments by combining the fine-tuned models with 30 new few-shot examples (excluded from the fine-tuning set) during evaluation. This hybrid approach offered marginal improvements, suggesting that prompt-based guidance can still help the fine-tuned models contextualize and refine their judgments. However, the overall findings reinforce that direct few-shot prompting outperforms fine-tuning in terms of evaluation accuracy, especially when computational resources allow for longer prompt lengths.

##### 4.4.3.3 Impact of contextual evaluation prompt routing

Given that few-shot prompting gives rise to long prompts and fine-tuning requires training large models on task-specific data, we experimented also with prompt routing which is a more scalable approach. Table 8 shows that even without few-shot examples, routing the prompts based on context improved the exact match accuracy from 0.742 to 0.894 for the LLM-based parser and from 0.485 to 0.909 for the rule-based parser for gemini-1.5-flash-002. When few-shot examples are included in the prompt, the accuracy further improved to 0.939 for both LLM-based parser and rule-based parser. This confirms that prompt routing is a highly effective approach for structured query parsing evaluation, even when applied to traditional rule-based parsing outputs.

These results suggest that prompt routing helps maintain prompt clarity while avoiding hallucination issues associated with long few-shot prompts.

##### 4.4.3.4 Scaling prompt routing to a larger dataset

Since Contextual Evaluation Prompt Routing yielded promising results, we evaluated its performance on a larger dataset that covers multiple search query categories. Up to this point, all the experiments had been conducted on the small-scale dataset consisting of 66 manually crafted queries. This small dataset was used to observe the effect of iterative improvements of the prompt on a controlled set of complex, long-form queries containing explicit filters and implicit keywords. By focusing on a small but diverse dataset, we were able to systematically determine the most effective evaluation approach for the problem. However, given the limited sample size, we need to validate the findings on a larger and more representative test set, ensuring the method's robustness across real-world search queries.

Table 10 presents the results on the large-scale dataset which consists of six categories with 100 queries in each category. The column labeled “Initial Few-Shot Prompt” gives the results using the refined prompt obtained in the small-scale dataset experiments. These results show how well the prompt optimized for a smaller manually curated dataset generalizes to a broader set of search queries.

**Table 10:** Contextual evaluation prompt routing evaluation results on large-scale dataset.

**Category**	**Initial few shot prompt**	**Improved few shot prompt**
Real estate	0.66	0.91
Vehicles	0.93	0.95
Used and brand new items	0.88	0.97
Vehicle parts, accessories and tuning	0.40	0.94
Other categories	0.94	0.95
No category	0.81	0.87

Evaluations are made by gemini-1.5-flash-002 model.

The small-scale dataset contains long and complex queries designed to test the system's ability to extract structured attributes effectively. However, the larger dataset was constructed from the most frequently searched queries on the online classified ads platform and thus the queries are mostly shorter and more ambiguous, creating a slight distributional shift between the two datasets. Due to this shift, evaluations on the large dataset using the original prompt revealed discrepancies in performance, highlighting the need for further refinements.

To address this, the prompt routing strategy was adjusted to better align with the characteristics of the larger dataset. The column labeled “Improved Few-Shot Prompt” represents the results obtained after modifying the prompts to accommodate for shorter and more ambiguous queries. These adjustments involved refining the category-specific few-shot examples and optimizing the evaluation instructions to account for real-world search behavior.

The largest performance improvement was observed in the *Vehicle Parts, Accessories & Tuning* category, where the accuracy increased from 0.40 to 0.94. Similarly, *Real Estate* queries saw a notable improvement from 0.66 to 0.91, indicating that category-specific prompt refinements significantly enhance the evaluation quality. However, *No Category* queries remained the most challenging with accuracy peaking at 0.87. This suggests that implicit category assignments are inherently harder to evaluate, as they rely more heavily on contextual inference rather than explicit query signals.

Overall, these findings confirm that prompt routing, particularly when combined with category-specific few-shot examples, provides a robust and scalable evaluation approach. The ability to adapt the prompts to different query distributions ensures that the LLM-as-a-Judge framework remains effective across diverse search environments.

##### 4.4.3.5 Reliability measuring

We conducted an experiment on the large-scale dataset to assess the reliability of the LLM-as-a-Judge system. For each category, we randomly selected five different subsets, each containing 20 samples. Agreement metrics were calculated across these subsets to capture the variability in LLM evaluations. Using these agreement scores, we computed the standard deviation (SD), coefficient of variation (CV), and mean absolute deviation (MAD) metrics.

Table 11 presents the variability in the LLM-based agreement scores across different categories. Lower values indicate more consistent evaluations across the subsets, while higher values suggest greater variability.

**Table 11:** Reliability scores across categories.

**Category**	**SD**	**CV (%)**	**MAD**
Real estate	0.042	4.358	0.050
Vehicles	0.060	6.433	0.072
Used and brand new items	0.048	5.082	0.058
Vehicle parts, accessories & Tuning	0.050	6.013	0.060
Other categories	0.075	7.711	0.067
No category	0.042	4.450	0.050

The results reveal that the *Real Estate* and *No Category* categories exhibit the lowest variability, implying that LLM evaluations in these domains are relatively stable. This is likely due to well-structured queries in the real estate sector and limited complexity in non-categoric queries, queries without any category assignment, such as “urgent.” In such queries, it is usually sufficient to extract only the explicit keyword, which simplifies the problem. Conversely, the *Other Categories* category demonstrates the highest variability, indicating significant inconsistencies in LLM evaluations across the subsets. This can be expected as the category aggregates diverse and sparsely represented queries, making structured evaluation more challenging.

### 4.5 Comparisons with related works

In the proposed approach, a search query is parsed into a structured form (search query parser output) that is used to retrieve the search results from a database. The quality of the structured output cannot be evaluated by executing it and counting the number of returned items, as a large set of results does not necessarily signal relevancy. For instance, omitting parsing altogether and performing a simple keyword search may yield a broad set of results, but many of them would not reflect the user's actual intent. In such a setup, the only reliable way to assess the quality of a parsed output is through human evaluation, examining whether the structured output accurately represents the user's semantic intent. However, manual evaluation of thousands of structured outputs is prohibitively time-consuming and costly. Therefore, we adopt the LLM-as-a-Judge framework as an efficient and semantically robust alternative. In assessing the performance of the proposed evaluation framework, we use correlation with human judgments as the evaluation metric. Traditional automated metrics (e.g., BLEU) or heuristic-based baselines are not directly applicable in our setting due to the nature of the search pipeline and the sparsity of ground truth labels at scale.

While our primary comparison is against expert human annotation on a small-scale validation set, we acknowledge the need to contextualize our results within the broader literature. Several recent studies have validated the effectiveness of LLM-based evaluation methods against human preferences and established their superiority over traditional baselines:

- Raju et al. (2024) introduce a domain-specific benchmark to evaluate LLMs as judges, achieving a Spearman correlation of 0.915 with human judgments, substantially outperforming existing baselines such as AlpacaEval 2.0 LC (0.297). Their study also reports 84% agreement with Chatbot Arena results, demonstrating that well-designed evaluation prompts tailored to the task domain can lead to highly reliable assessments.
- TALEC (Zhang et al., 2024) proposes a framework for training LLM evaluators with task-specific criteria, reporting Spearman correlations of 0.96–0.97 for tasks such as sentiment analysis and title generation. The study also highlights that average correlation with human judgment exceeds 0.80, often surpassing inter-human agreement in subjective tasks.
- Zheng et al. (2023), use the LLM-as-a-Judge strategy with MT-Bench and Chatbot Arena and report that GPT-4 achieves 85% agreement with human experts, which is higher than the 81% agreement among human annotators themselves. This reinforces the reliability of advanced LLMs like GPT-4 as scalable surrogates for human judgment in evaluation pipelines.

In our study, we similarly observe around 90% agreement between the LLM-based evaluations and human annotations using different prompt configurations. These results are in line with the findings in the literature and confirm that LLM-as-a-Judge can serve as a reliable and scalable alternative to manual evaluation for structured output tasks in search systems.

Finally, we emphasize that while real-time A/B testing metrics such as click-through rate (CTR) and exit rate will eventually serve as automated feedback signals in production, these signals are not currently accessible during the offline development phase. As such, traditional automated baselines cannot be employed to evaluate the effectiveness of query parsing outputs at scale prior to deployment. In this setting, LLM-as-a-Judge serves as an indispensable evaluation mechanism, offering a scalable and semantically grounded alternative to manual annotation.

### 4.6 Error analysis

In this section, we briefly mention some error cases and limitations of the proposed framework. One issue is the tendency of the search query parser to generate hallucinated outputs. The parser may assign categories that do not exist in the predefined category taxonomy or it may fail to make category assignments at the appropriate hierarchical level and assigns categories at a more granular level than required. Although LLM-as-a-Judge is generally effective in assessing the accuracy of the structured outputs, it exhibits improper evaluations in cases where such hallucinations occur. The main reason for these errors is that LLM-as-a-Judge lacks reference ground truth values, relying solely on the instructions provided in the system prompt of the search query parser. When the prompt does not contain sufficiently detailed explanations, the evaluation process becomes susceptible to errors as the model has no alternative means of verifying correctness.

Another limitation of the framework involves domain-specific search requirements. In cases of incorrect category matching, the model for instance may misclassify a term such as “golf” under sports rather than identifying it as a car model (Volkswagen Golf). In such cases, LLM-as-a-Judge struggles to detect misclassifications, leading to erroneous evaluations. Furthermore, the model exhibits difficulty in recognizing implicit information within the search queries. For example, the word “paint-free” implies that a vehicle is undamaged and the filters should be extracted according to this. However, the search query parser fails to infer this meaning and LLM-as-a-Judge does not correctly flag the response as erroneous.

These findings suggest that enhancing the system prompt with more detailed explanations and incorporating domain-specific knowledge 5improve the reliability of LLM-as-a-Judge in evaluating search query parser outputs.

## 5 Conclusion

In this paper, we introduced LLM-as-a-Judge, a general framework for evaluating structured outputs, with a specific focus on search query parsing in an online classifieds platform. Unlike traditional evaluation methods, the proposed approach leverages LLMs' reasoning abilities to assess structured outputs more effectively, ensuring context-aware, interpretable, and scalable evaluations. We proposed three evaluation methodologies, Pointwise, Pairwise, and Pass/Fail, to cover different assessment needs, and we further enhanced reliability and efficiency with the Contextual Evaluation Prompt Routing strategy which dynamically adjusts evaluation prompts based on query categories. To validate the framework, we conducted experiments on two datasets which are a small, manually curated dataset and a large, real-world dataset. The small dataset enabled us to iteratively refine our evaluation prompts and methodologies, while the large dataset allowed us to test the scalability of the prompt routing approach. The findings confirmed that routing the prompts based on context of the query significantly improves the evaluation accuracy, particularly in category-specific few-shot prompting. Also, the reliability analysis showed that this approach is highly effective for well-defined, high-traffic categories, while more ambiguous queries require further optimization to achieve consistent evaluations.

The experimental results highlighted key insights into the performance of different evaluation techniques, some of which are outlined below:

- Pointwise evaluation: across both LLM-based and rule-based parser outputs, few-shot prompting generally improved alignment with human scores, as reflected by increased Spearman's correlation. For example, in the rule-based parser, correlation rose from 0.564 to 0.793 (flash-002) and from 0.677 to 0.853 (pro-002). Incorporating reference values further boosted alignment, achieving correlation scores of up to 0.898 for the LLM-based parser and 0.870 for the rule-based parser.
- Pairwise evaluation: by adding few shot examples to the prompt we improved the alignment from 0.773/0.758 to 0.879 for both models. Also, we mitigated position bias by randomizing the order of the outputs of the two parsers, improving exact match accuracy from 0.833 to 0.879.
- Pass/fail evaluation: the prompt routing method achieved 0.939 exact match accuracy and 0.861 Cohen's Kappa (flash-002) on the small-scale dataset and 0.87–0.97 exact match accuracy on the large-scale real world dataset for the LLM-based parser, demonstrating its effectiveness in binary classification tasks.

As future work, we aim at improving the reliability of the evaluation of ambiguous queries, specifically those without a clear category and those outside the four main categories. This will involve expanding the prompt diversity by incorporating a broader range of query formulations and optimizing prompt routing strategies for these underrepresented categories. Additionally, we plan to explore alternative LLM architectures and fine-tune the models with domain-specific evaluation data to further enhance alignment with human judgments. Another key direction is automating the refinement process by iteratively identifying failure cases and adapting the evaluation prompts dynamically. Also, we will investigate cross-domain generalization, applying the LLM-as-a-Judge framework to other structured output evaluation tasks beyond search query parsing. Lastly, once the system is live in the future, we plan to use live A/B testing metrics as a post-deployment baseline to measure the practical effectiveness of the LLM-as-a-Judge evaluations. These real-world metrics will allow us to retrospectively validate and calibrate the judgments made by our LLM-based evaluation framework, providing a closed-loop mechanism that combines human-aligned semantic assessment with behavioral user signals from production.

## Data Availability

The datasets presented in this article are not readily available because the search datasets are at the proprietary of the company, we cannot make the datasets publicly available. Requests to access the datasets should be directed to mehmet.baysan@sahibinden.com.

## Footnotes

^1^Since the datasets are at the proprietary of the company, we cannot make the datasets publicly available.

^2^Google Gemini: https://deepmind.google/technologies/gemini/, Last Access Date: 07.04.2025.
