# Supplementary material for: LLM-as-a-Judge: automated evaluation of search query parsing using large language models
Source: Front Big Data. 2025 Jul 21;8:1611389. doi: 10.3389/fdata.2025.1611389 (PMC12319771; doi:10.3389/fdata.2025.1611389)
Supplement: Supplementary file 1 [file Data_Sheet_1.pdf]

Supplementary Material

1 SUPPLEMENTARY DATA

1.1 Pointwise Prompt

```
# Instruction
You are an expert evaluator assessing the performance of Large Language Models (LLMs) in extracting
    structured information from unstructured search queries.  Your task is to evaluate the accuracy and
    completeness of the LLM's JSON response in representing the user's search intent.  The evaluation
    will focus on the extraction of categories, location, filters, and keywords, both explicit and
    implicit.  Refer to the provided category and filter trees (provided in the prompt) to assess the
    correctness of the LLM's categorization.


# Evaluation
## Metric Definition
This metric assesses the accuracy and completeness of the LLM's structured representation of a user's
    search query.  A perfect score indicates a complete and accurate extraction of all relevant
    information.

## Criteria
{given in Table 1}


## Rating Rubric
{given in Table 2}


## Evaluation Steps
STEP 1: Verify Category Accuracy: Check each category level against the provided category tree.
STEP 2: Verify Filter Accuracy: Examine each filter for accuracy and completeness.  Pay close attention
    to numerical values and boolean flags.
STEP 3: Verify Location Accuracy:  Check if the location information is correctly extracted or
    appropriately marked as null if not present in the query.
STEP 4: Verify Keyword Accuracy:  Assess both explicit and implicit keyword extraction. Consider the
    context of the search query.
STEP 5: Verify Synonym Accuracy: Examine the synonyms for each keyword, ensuring relevance and accuracy.
STEP 6: Assess Overall Completeness: Evaluate the completeness of the JSON response; are all expected
    fields present?


# Examples: (This section is included in the evaluation prompt when the few-shot flag is on. General
    examples are given to cover each category and filter condition.)
You can refer to the examples below to understand the evaluation criteria better.

##Example 1:
...


# User Inputs and AI-generated Response
## User Inputs
```

```
### Prompt
{Search Query Parser Prompt}

## AI-generated Response
{response}
```

1.2 Pairwise Prompt

```
# Instruction
You are an expert evaluator. Your task is to evaluate the quality of responses generated by two AI
    models for a given user search query. You will be presented with two different LLM responses (JSON
    format) - Response A and Response B.

**First, carefully analyze the user query to understand the search intent.** Develop your own ideal
    JSON response based on the query and the provided category/filter tree. This will serve as your
    benchmark for evaluating the LLM responses.

**Next, evaluate each response (A and B) individually against the following criteria, comparing them to
    your ideal response:**

## Criteria:
{given in Table 1}


## Rating Rubric:
{given in Table 3}


## Evaluation Steps:
1. **Understand the Query:** Carefully analyze the user query and determine the search intent.
2. **Formulate Ideal Response:** Construct your own ideal JSON response based on the query, considering
    the provided category/filter tree.
3. **Evaluate Response A:** Compare Response A to your ideal response, assessing each criterion.
4. **Evaluate Response B:** Compare Response B to your ideal response, assessing each criterion.
5. **Compare and Explain:** Compare the performance of Response A and B based on your evaluations
    against your ideal response. Provide a detailed explanation justifying your final choice.
6. **Final Decision:** Output your preference of \"A\", \"SAME\", \"B\", or \"UNCLEAR\" to the
    ‘pairwise_choice‘ field according to the Rating Rubric. Be particularly discerning when choosing
    \"UNCLEAR\", ensuring it reflects genuine inadequacy in both responses compared to your ideal
    response.

# Examples: (This section is included in the evaluation prompt when the few-shot flag is on. General
    examples are given to cover each category and filter condition.)
You can refer to the examples below to understand the evaluation criteria better.

##Example 1:
...

# User Inputs and AI-generated Responses
## User Inputs
### Prompt
{Search Query Parser Prompt}

# AI-generated Responses
```

```
### Response A
{response_a}

### Response B
{response_b}
```

1.3 Pass Fail Prompt

```
# Instruction
You are an expert evaluator. Your task is to evaluate the quality of a single AI-generated response (in
    JSON format) for a given user search query. Your goal is to determine if the response is valid and
    accurately captures the user's search intent.

**First, carefully analyze the user query to understand the search intent.** Develop your own ideal
    JSON response based on the given system prompt and the provided category/filter tree inside the
    system prompt. This ideal response will serve as your benchmark for evaluating the LLM response.

**Next, evaluate Response against your ideal response using the following criteria:**

## Criteria:
{given in Table 1}


## Rating Rubric:
{given in Table 4}


## Evaluation Steps:
1. **Understand the Query:** Carefully analyze the user query to determine the search intent.
2. **Formulate Ideal Response:** Construct your own ideal JSON response.
3. **Evaluate Response A:** Compare response to your ideal response using the criteria above.
4. **Final Decision:** Output your final decision ("PASS" or "FAIL") based on the Rating Rubric.

# Examples: (This section is included in the evaluation prompt when the few-shot flag is on. General
    examples are given to cover each category and filter condition.)
You can refer to the examples below to understand the evaluation criteria better.

##Example 1:
...

# User Inputs and AI-generated Responses
## User Inputs
### Prompt
{Search Query Parser Prompt}

# AI-generated Response
### Response
{response}
```

1.4 Contextual Evaluation Prompt Routing

1.4.1 Real Estate Evaluation Prompt

```
# Instruction
```

```
You are an expert evaluator. Your task is to evaluate the quality of a single AI-generated response (in
    JSON format) for a given user search query. Your goal is to determine if the response is valid and
    accurately captures the user's search intent.

**First, carefully analyze the user query to understand the search intent.

**You may develop your own ideal JSON response based on the given system prompt and the provided
    category/filter tree inside the system prompt. This ideal response will serve as your benchmark for
    evaluating the LLM response.

**Next, evaluate llm response against your ideal response using the following criteria:**

## Criteria:
{given in Table 1}

## Domain Specific Criteria
{Several criteria related to Real Estate domain listed below, sample criteria is below}
...
!!! If you see queries like "For Sublease" etc. that implies a business place to take over, it should
    be categorized under "Real Estate" -> "Business Place" -> "Rental" instead of "Real Estate" ->
    "Timeshare Property". So if you see another categorization, it should be considered as a failure.
    !!!
...

## Rating Rubric:
{given in Table 4}

## Evaluation Steps:
1. **Understand the Query:** Carefully analyze the user query to determine the search intent.
2. **Formulate Ideal Response:** Construct your own ideal JSON response.
3. **Evaluate Response:** Compare response to your ideal response using the criteria above.
4. **Decision:** Output your decision ("PASS" or "FAIL") based on the Rating Rubric.

# Examples: (This section is included in the evaluation prompt when the few-shot flag is on. Examples
    are given to cover only the filter and category conditions specific to this Real Estate
    subcategory.)
You can refer to the examples below to understand the evaluation criteria better.

## Example 1:
### Question:
If user search '3+1 apartment for sale with balcony in the complex', return parsed query in json format

### Response:
{{
    "category_level_1": "Real Estate",
    "category_level_2": "Housing",
    "category_level_3": [
        "For Sale"
    ],
    "category_level_4": [
        "Apartment"
    ],
    "location": {{
        "Province": null,
```

```
        "District ": null
    }},
    "filters": {{
        "Number of Rooms": [
            "3+1"
        ],
        "In a Complex": "Yes",
        "Balcony": "Yes"
    }},
    "explicit_keywords": [],
    "implicit_keywords": [],
    "synonym_keywords": []
}}


### Explanation:
Response correctly identifies the category levels, filters (including "Balcony": "Yes" although it
    might not mentioned in the filter tree in the system prompt, it is correctly derived from "with
    balcony" phrase), and keywords. It accurately reflects the user's search intent.

## Decision:
PASS

## Example 2:
### Question:
Question:
If user searches for "land with rental income", return parsed query in JSON format.

Response:
{
  "category_level_1": "Real Estate",
  "category_level_2": "Land",
  "category_level_3": ["For Rent"],
  "category_level_4": null,
  "location": {
    "province": null,
    "district": null
  },
  "filters": {
    "Price": {
      "min": null,
      "max": null
    }
  },
  "explicit_keywords": ["rental", "income", "land"],
  "implicit_keywords": [],
  "synonym_keywords": []
}

Explanation:
The AI incorrectly interpreted the search term  land  with rental income  as a request to rent a
    land parcel. However, the user is more likely intending to buy land that generates rental income.
Therefore, the appropriate category structure should be "Real Estate"    "Land"    "For Sale",
```

```
and the phrase  land  with rental income should remain part of the explicit keywords, rather than
    being interpreted as an indicator for the rental category.

Decision:
FAIL

...

# User Inputs and AI-generated Responses
## User Inputs
### Prompt
{Search Query Parser Prompt}

# AI-generated Response
### Response
{response}
```

1.5 Full Experiment Results

1.5.1 Pointwise Experiment Results

1.5.2 Pairwise Experiment Results

1.5.3 Pass/Fail Experiment Results

Table S1. Pointwise Evaluation Results for LLM-based Parser Outputs

Evaluator Model	Prompt Type	Spearman's Correlation	Standard Deviation Across Runs
gemini-1.5-pro-001	Basic Prompt	0.380	0.443
	Basic Prompt + Few Shot	0.478	0.460
	Basic Prompt + Few Shot + Explain First	0.531	0.473
	Basic Prompt + Few Shot + Separate System Prompt	0.350	0.337
	Basic Prompt + Few Shot + Reference Values	0.844	0.277
gemini-1.5-pro-002	Basic Prompt	0.490	0.061
	Basic Prompt + Few Shot	0.445	0.464
	Basic Prompt + Few Shot + Explain First	0.461	0.453
	Basic Prompt + Few Shot + Separate System Prompt	0.354	0.402
	Basic Prompt + Few Shot + Reference Values	0.858	0.209
gemini-1.5-flash-001	Basic Prompt	0.203	0.139
	Basic Prompt + Few Shot	0.542	0.072
	Basic Prompt + Few Shot + Explain First	0.101	0.113
	Basic Prompt + Few Shot + Separate System Prompt	0.324	0.005
	Basic Prompt + Few Shot + Reference Values	0.768	0.080
gemini-1.5-flash-002	Basic Prompt	0.381	0.023
	Basic Prompt + Few Shot	0.364	0.023
	Basic Prompt + Few Shot + Explain First	0.350	0.184
	Basic Prompt + Few Shot + Separate System Prompt	0.565	0.020
	Basic Prompt + Few Shot + Reference Values	0.898	0.024

Table S2. Pointwise Evaluation Results for Rule-based Parser Outputs

Evaluator Model	Prompt Type	Spearman's Correlation	Standard Deviation Across Runs
gemini-1.5-pro-001	Basic Prompt	0.905	0.569
	Basic Prompt + Few Shot	0.847	0.492
	Basic Prompt + Few Shot + Separate System Prompt	0.878	0.366
	Basic Prompt + Few Shot + Reference Values	0.902	0.396
gemini-1.5-pro-002	Basic Prompt	0.677	0.062
	Basic Prompt + Few Shot	0.853	0.508
	Basic Prompt + Few Shot + Separate System Prompt	0.711	0.458
	Basic Prompt + Few Shot + Reference Values	0.832	0.492
gemini-1.5-flash-001	Basic Prompt	0.138	0.135
	Basic Prompt + Few Shot	0.244	0.271
	Basic Prompt + Few Shot + Separate System Prompt	0.252	0.000
	Basic Prompt + Few Shot + Reference Values	0.797	0.126
gemini-1.5-flash-002	Basic Prompt	0.564	0.082
	Basic Prompt + Few Shot	0.793	0.057
	Basic Prompt + Few Shot + Separate System Prompt	0.785	0.015
	Basic Prompt + Few Shot + Reference Values	0.870	0.007

Table S3. Pairwise Evaluation Results

Evaluator Model	Prompt Type	Exact Match Accuracy	Cohen's Kappa
gemini-1.5-flash-001	Basic Prompt	0.727	0.569
	Basic Prompt + Few Shot (Initial)	0.697	0.535
	Basic Prompt + Few Shot (Final)	0.742	0.603
gemini-1.5-flash-002	Basic Prompt	0.773	0.635
	Basic Prompt + Few Shot (Initial)	0.773	0.632
	Basic Prompt + Few Shot (Final)	0.879	0.807
	Prompt Routing	0.697	0.508
	Prompt Routing + Few Shot (Initial)	0.758	0.607
	Prompt Routing	0.402	0.092
	Prompt Routing + Few Shot (Initial)	0.664	0.081
gemini-1.5-pro-001	Basic Prompt	0.727	0.568
	Basic Prompt + Few Shot (Initial)	0.727	0.584
	Basic Prompt + Few Shot (Final)	0.758	0.634
gemini-1.5-pro-002	Basic Prompt	0.758	0.610
	Basic Prompt + Few Shot (Initial)	0.758	0.610
	Basic Prompt + Few Shot (Final)	0.879	0.816
cline2-4	Prompt Routing	0.742	0.583
	Prompt Routing + Few Shot (Initial)	0.803	0.687
	Prompt Routing	0.481	0.181
	Prompt Routing + Few Shot (Initial)	0.671	0.151

Table S4. Pass/Fail Evaluation Results for LLM-based Parser Outputs

Evaluator Model	Prompt Type	Exact Match Accuracy	Cohen's Kappa
gemini-1.5-flash-001	Basic Prompt	0.718	0.115
	Basic Prompt + Few Shot (Initial)	0.727	0.223
	Basic Prompt + Few Shot (Final)	0.909	0.779
	Prompt Routing	0.803	0.571
	Prompt Routing + Few Shot (Initial)	0.909	0.796
gemini-1.5-flash-002	Basic Prompt	0.742	0.282
	Basic Prompt + Few Shot (Initial)	0.727	0.250
	Basic Prompt + Few Shot (Final)	0.848	0.659
	Prompt Routing	0.894	0.747
	Prompt Routing + Few Shot (Initial)	0.939	0.861
gemini-1.5-pro-001	Basic Prompt	0.742	0.370
	Basic Prompt + Few Shot (Initial)	0.789	0.462
	Basic Prompt + Few Shot (Final)	0.788	0.462
	Prompt Routing	0.788	0.475
	Prompt Routing + Few Shot (Initial)	0.894	0.759
gemini-1.5-pro-002	Basic Prompt	0.727	0.250
	Basic Prompt + Few Shot (Initial)	0.727	0.270
	Basic Prompt + Few Shot (Final)	0.848	0.625
	Prompt Routing	0.818	0.526
	Prompt Routing + Few Shot (Initial)	0.909	0.780

Table S5. Pass/Fail Evaluation Results for Rule-based Parser Outputs

Evaluator Model	Prompt Type	Exact Match Accuracy	Cohen's Kappa
gemini-1.5-flash-001	Basic Prompt	0.576	0.194
	Basic Prompt + Few Shot (Initial)	0.852	0.704
	Basic Prompt + Few Shot (Final)	0.867	0.757
	Prompt Routing	0.712	0.405
	Prompt Routing + Few Shot (Initial)	0.818	0.629
gemini-1.5-flash-002	Basic Prompt	0.485	0.040
	Basic Prompt + Few Shot (Initial)	0.894	0.787
	Basic Prompt + Few Shot (Final)	0.803	0.593
	Prompt Routing	0.909	0.816
	Prompt Routing + Few Shot (Initial)	0.939	0.878
	Prompt Routing	0.371	0.127
	Prompt Routing + Few Shot (Initial)	0.309	0.102
gemini-1.5-pro-001	Basic Prompt	0.758	0.504
	Basic Prompt + Few Shot (Initial)	0.803	0.586
	Basic Prompt + Few Shot (Final)	0.848	0.681
	Prompt Routing	0.742	0.463
	Prompt Routing + Few Shot (Initial)	0.833	0.653
gemini-1.5-pro-002	Basic Prompt	0.848	0.692
	Basic Prompt + Few Shot (Initial)	0.909	0.815
	Basic Prompt + Few Shot (Final)	0.955	0.907
	Prompt Routing	0.773	0.544
	Prompt Routing + Few Shot (Initial)	0.909	0.816
	Prompt Routing	0.802	0.497
	Prompt Routing + Few Shot (Initial)	0.815	0.186
